# Gender-Based Differences on the Association between Salt-Sensitive Genes and Obesity in Korean Children Aged between 8 and 9 Years

**DOI:** 10.1371/journal.pone.0120111

**Published:** 2015-03-13

**Authors:** Myoungsook Lee, Mi Kyung Kim, Seon-Mee Kim, Hyesoon Park, Chang gyu Park, Hye Kyung Park

**Affiliations:** 1 Department of Food and Nutrition, and Research Institute of Obesity Sciences, Sungshin Women’s University, Seoul, Republic of Korea; 2 Department of Epidemiology, National Cancer Center, Ilsan, Republic of Korea; 3 Division of Family Medicine, Department of Internal Medicine, Korea University Guro Hospital, Seoul, Republic of Korea; 4 Department of Family Medicine, School of Medicine, Ulsan University Asan Hospital, Seoul, Republic of Korea; 5 Division of Cardiovascular diseases, Department of Internal Medicine, Korea University Guro Hospital, Seoul, Republic of Korea; 6 Division of Nutrition policy, Korean Food and Drug Administration, Cheongju, Republic of Korea; University Medical Center Utrecht, NETHERLANDS

## Abstract

**Background:**

High sodium intake is associated with the development of chronic diseases such as obesity. Although its role in obesity remains controversial, there may be a correlation between salt sensitivity and the early onset of chronic diseases in obese children.

**Methods:**

In all, 2,163 Korean children (1,106 boys and 1,057 girls) aged 8–9 years were recruited from seven elementary schools in Seoul. To evaluate whether obesity risk was modulated by the salt sensitivity, 11 SNPs related to salt sensitive genes (SSG) became the target of sodium intakes in obese children.

**Results:**

BP, HOMA-IR, LDLc, TG, and the girls’ sodium intake significantly increased, but HDLc significantly decreased with increase in BMI. Regardless of sex, the obesity risk was 5.27-fold (CI; 1.320–27.560) higher in the Q2 to Q5 of sodium intake adjusted by energy (4044.9–5058.9 mg/day) than in the lowest Q1 level (2287.6 mg/day) in obese children. BP was sensitively dependent on insulin resistance and lipid accumulation in all subjects; however, sodium intake may be an independent risk factor of obesity without increasing BP in girls. *GRK4 A486V* mutant homozygote was highly distributed in the obese group, but other SNPs had no impact. The obesity risk increased 7.06, 16.8, and 46.09-fold more in boys with *GRK4 A486V*, *ACE*, and *SLC12A3* mutants as sodium intake increased. Among girls, the obesity risk increased in *GRK4 A486V* heterozygote and *CYP11β-2* mutant homozygote although sodium intake was relatively lower, implying that *ACE*, *SLC12A*, *CYP11β-2*, and *GRK4 A486V* polymorphisms showed gender-based differences with regard to interaction between sodium intake and obesity.

**Conclusion:**

A high sodium intake markedly increased the obesity risk in variants of *GRK4 A486V* regardless of sex. The obesity risk increased with *GRK4 A486V*, *ACE*, and *SLC12A3* variants in boys, whereas it increased with *GRK4 A486V* and *CYP11B2* variants in girls as sodium intake increased. Obese children with the specific gene variants are recommended to reduce their sodium intake.

## Introduction

According to the 2010 Korean National Health and Nutrition Examination Survey (KNHANES), the average daily sodium intake (5000 mg/day; dietary salt intake 13.8 g/day) of Korean adults is 2.5-fold higher than that recommended by the WHO.[1] The role of dietary sodium in the development of hypertension, obesity, renal, and cardiovascular disease is still debatable. Salt sensitivity and hypertension are complex diseases that may be the result of genetic predisposition coupled with environmental influences, such as excessive sodium consumption and sedentary lifestyles. Even though there was considerable controversy about the definition and diagnosis of salt sensitivity, the most reliable method to measure salt sensitivity is the blood pressure (BP) response to a change in dietary sodium intake as shown in epidemiologic studies. [2,3,4] In a Chinese study on sodium intervention, multivariable-adjusted mean changes in BP were significantly greater in subjects with metabolic syndrome than in those without it, among both those consuming low-sodium (51.3 mM/day) and high-sodium (307.8 mM/day) diets. [5] This finding implies that the reduction in sodium intake could be an important component in patients with multiple risk factors for metabolic syndrome, including obesity.

Since half of the 70 million Americans with high BP who were keeping it under control by medication are obese, obesity may be associated with high BP, and vice versa. [6] Several studies have shown that obese rodent models also have impaired pressure-natriuresis and increased salt sensitivity, where the association between salt sensitivity and obesity requires investigation. [7,8] According to the cytokine-based hypertension hypothesis, inflammation factors, IL-6 or c-reactive protein, were released by fat accumulation, activating the epithelial sodium channel (*ENaC*), SSG, and increasing sodium reabsorption in the kidney.[9] Other researchers have suggested that the salt sensitivity was mediated by insulin, leptin resistance, or urinary sodium excretion regulated by 5′AMP-activated protein kinase (AMPK), an energy regulator.[10,11] Many papers have reported that the variation in BP environments are due to the combined effects of SSG such as angiotensin-converting enzyme *(ACE)*, angiotensinogen *(AGT)*, α-adducin1 *(ADD1)*, cytochrome P450 family 11-subfamily β-2 *(CYP11β-2)*, G-protein b3 subunit *(GNB3)*, *G* protein-coupled receptor kinases type 4 *(GRK4 A142V*, *GRK4 A486V)*, 11β-hydroxysteroid dehydrogenase type-2 *(HSD 11β-2)*, neural precursor cell-expressed developmentally downregulated 4 like *(NEDD4L)*, solute carrier family 12 (sodium/chloride transporters)-member 3 *(SLC 12A3)*, and *ENac*, and may modulate the risk of obesity.[4,12,13,14] However, there is no information regarding the effect of sodium intake on white adipose tissue lipogenic and lipolytic fluxes. However, the mechanisms how the polymorphisms of SSG develop the obesity in humans still remains unclear. The purpose of this study was to investigate the association of obesity risk in Korean children with a dietary habit of high sodium intake. We also investigated the polymorphism of SSG that may be involved in the risk of child obesity in spite of diversity among different ethnic groups. We believe that the findings of this study will contribute to the prevention of the early onset of chronic disease in obese children, facilitating personalized management of obesity from childhood to adulthood.

## Material and Methods

### Subjects

In all, 2,163 subjects (8 and 9 years of age; 1,106 boys and 1,057 girls) were recruited from the 3^rd^ grade from seven elementary schools located Guro-ku in Seoul, Korea, in the period of regular medical checkups at 2007 (n = 1,128) and 2008 (n = 1,035). All participants were examined using a basic survey encompassing their parent’s health history, BMI, education and income, physical activity, and dietary sodium intake. This study protocol was approved by the institutional review board of Korea University, Guro Hospital (#:GR0837–001) and a written informed consent was obtained from the children and their parents.

### Anthropometry and Blood Biochemistry

Height (Ht), body weights (BW), and waist circumference (WC, cm) were measured, and body mass index [BMI = BW (kg)/Ht (m^2^)] and obesity index [OI, % = 100+ (present BW—ideal BW)/ideal BW×100)] were calculated. Overweight was defined if the BMI was between the 85th and 90th percentile, OI> 110%, and WC was between the 50th percentile and 75th percentile. Obesity was defined if BMI >95th percentile, OI >120%, and WC > 75th percentile. Blood samples were obtained to measure total cholesterol (TC), triglyceride (TG), high density lipoprotein cholesterol (HDLc) and fasting blood glucose (FBS) using a Hitachi-7600 analyzer (Hitachi, Tokyo, Japan). Low-density lipoprotein cholesterol (LDLc) levels were calculated using the equation: LDLc = TC–HDLc–(TG/5). Commercial ELISA kits were used for estimating fasting insulin levels (Mesdia, Seoul, Korea). The homeostasis model assessment of insulin resistance (HOMA-IR) was calculated as follows: fasting insulin (μU/ml)×FBS (mmol/ml)/22.5. We genotyped nine SNPs polymorphisms using the TaqMan Allelic Discrimination method (SDS 7700 ABI, Applied Biosystems Inc., Foster City, CA, USA) for *GNB3(rs5443);* ultra-high throughput method for *SCL12A3(rs116437818)*, *CYP11β-2(rs1799998)*, *GRK4 A142V (rs1024323)*, *GRK4 A486V (rs1801058)*, *AGT(rs699)*, *ACE (rs4341)*, and *NEDD4L(rs2288774);* and SNaPShot method for *ADD1(rs4961)*.[15,16,17] (S1 Table). The three genotypes of each SNP were described as wild for dominant or major homozygotes, mutant for recessive or minor homozygotes, and heterozygote.

### Dietary Intake of Sodium

Due to the constraint of the participants’ young age, dietary intake, including that of sodium, were assessed by performing the three-day 24-h food recall survey instead of the 24-hr urinary excretion method. Dietary intake for three days (two weekdays and one weekend day) was recorded by a trained interviewer and the food records were crosschecked with their parents or guardians. In all, food records for 2,163 subjects were finally included in the dietary analysis after excluding incomplete, undetectable, or unreliable records. CAN-Pro 4.0 (Korean Nutritional Society, Seoul, Korea) was used for the quantitative analysis of nutrients based on food records.

### Statistical Analysis

All analyses were performed using SAS 9.1 software (SAS Institute, Inc., Cary, NC, USA). All statistical tests were two-tailed, and *p-* values of ≤0.05 were interpreted as statistically significant. The χ^2^ test was used to compare the differences in proportions of covariates between the cases and control subjects. The mean and standard deviations were calculated for continuous demographic variables with the mean differences tested with adjustments for age by ANOVA or ANCOVA (age-adjusted). Tests for Hardy–Weinberg equilibrium for each SNP among the controls were conducted by comparing the observed and expected genotype frequencies of the controls with ***X*** ^2^ test with one degree of freedom. Logistic regression models (PROC LOGISTIC, SAS Version 9.1; SAS Institute, Cary, NC) were used to estimate odds ratios (ORs) and corresponding 95% confidence intervals (CIs). Multivariate logistic regression models was performed to evaluate the association of each SNP with the risk of obesity after adjustment for age, sex, BMIs and education levels (<10 years, 10~12, 13~16 years, and >16 years) of the mother and father and regular physical activity (no, yes). Linear trends were calculated using the median values for each risk factor as a continuous variable. Energy-adjusted sodium intake was used by residual methods and categorized into quintiles based on the distribution of sodium intake in all subjects.[18] Multivariate OR and CI for obesity risk by quintiles of energy-adjusted sodium intake was computed after adjustment for age, sex, mother’s BMI, father’s BMI, and educational experience (<10 years, 10~12, 13~16 years, and >16 years) of the mother and father, dietary fat, systolic blood pressure, and regular physical activity (no, yes). We evaluated the association of obesity with dietary sodium intake according to each SNP. Interaction tests were performed using multiplicative interaction terms of the ordinal score for quintiles of energy-adjusted sodium intake and each SNP of several genes in the multivariate logistic regression models after adjustment for age, sex, mother’s BMI, father’s BMI, and education levels (<10 years, 10~12, 13~16 years, and >16 years) of the mother and father, dietary fat, systolic blood pressure and regular physical activity (no, yes). Variables for joint effects were coded using the lowest quintile of energy-adjusted sodium intake with a wild type of each SNP used as a reference group.

## Results

### General Characteristics of Children

A difference in obesity distribution was found in the boys who were classified as normal, overweight, and obese. There were no differences in obesity distribution by age among the girls (Table 1). Blood pressure (SBP/DBP), insulin, HOMA-IR, LDL, TG, and the TG/HDL ratio significantly increased in both sexes, but the HDL significantly decreased as the children’s obesity increased. There was no difference in the FBS level between the non-obese and obese children. However, insulin and HOMA-IR levels were approximately 2.5-fold higher in the obese children. Furthermore, exercise was shown to be uncorrelated with obesity in the children. This was attributable to the fact that the children generally take an exercise for one to three hours every day. The obese children seemed to have inherited obesity as judged by the BMI of their parents, but it was not correlated with parents’ education and income, health supplement intake, time of TV watching, and snack intake (data not shown). As for the causal relationship with diet, obesity was not correlated with the total intake for energy, sugar, protein, and fats. In obese girls, sodium intake was 3926.6± 1259.3 mg/day, which was significantly greater by more than 500 mg/day, compared to normal girls (p = 0.03). BP was sensitively dependent on the effects of insulin resistance on lipid accumulation in all subjects. However, in obese girls, we concluded that sodium intake could be the risk factor for obesity-induced insulin resistance, independent of BP, because BP and blood insulin, HOMA-IR, TG and LDL levels were not increased by high sodium intake.

**Table 1:** General characteristics of subjects in this study.

Characteristics^6^	Total (n = 2163)				Boy (n = 1106)				Girl (n = 1057)			
	Normal	Overweight	Obestiy^5^	p-value^4^	Normal	Overweight	Obestiy^5^	p-value^4^	Normal	Overweight	Obestiy^5^	p-value^4^
**Subject(n)**	1,769	309	85		875	175	56		894	134	29	
**Age (years)**												
8	43.53^1^	41.75	40.7	NS^2^	35.71	3.14	39.54	NS^2^	58.62	47.76	41.83	NS^2^
9	52.94	55.02	59.13		58.93	57.71	60.23		41.38	51.49	58.05	
**Blood markers**												
SBP	107.02(15.33)^3^	117.31(19.42)	122.29(18.20)	<0.001	108.03(15.85)	119.52(19.44)	122.49(17.97)	<0.001	121.92(18.96)	114.65(19.13)	106.04(14.74)	<.0001
DBP	68.42(12.48)	74.95(14.55)	78.05(14.25)	<0.001	68.54(12.45)	76.03(14.49)	78.51(14.60)	<0.001	77.23(13.83)	73.65(14.58)	68.31(12.53)	<.0001
FBS	78.96(9.90)	80.49(12.04)	78.75(8.83)	NS	79.62(10.66)	81.39(13.28)	78.72(8.13)	NS	78.81(10.31)	79.65(10.16)	78.31(9.04)	NS
Insulin	6.32(5.43)	9.00(5.03)	14.18(13.00)	<0.001	6.25(5.66)	8.74(5.35)	13.01(11.70)	<0.001	16.37(15.16)	9.31(4.61)	6.38(5.19)	<.0001
HOMA-IR	43.81(37.70)	62.51(34.91)	98.46(90.31)	<0.001	43.34(39.32)	60.70(37.12)	90.37(81.28)	<0.001	113.70(105.29)	64.69(32.04)	44.27(36.05)	0.0024
HDL	57.09(10.17)	52.61(9.43)	49.57(9.12)	<0.001	58.22(10.17)	53.39(9.49)	49.93(9.58)	<0.001	48.82(8.21)	51.60(9.30)	55.97(10.08)	<.0001
LDL	100.28(26.73)	108.61(26.42)	111.73(27.65)	<0.001	98.19(25.79)	107.17(27.62)	111.91(24.86)	<0.001	111.36(33.26)	110.46(24.77)	102.33(27.48)	0.0024
TG	70.80(36.10)	94.80(54.91)	107.56(53.23)	<0.001	66.15(35.53)	86.59(51.87)	102.74(45.43)	<0.001	117.58(66.51)	105.40(57.07)	75.39(36.08)	<.0001
TG/HDL	1.33(0.88)	1.95(1.39)	2.34(1.48)	<0.001	1.22(0.87)	1.74(1.25)	2.22(1.29)	<0.001	2.59(2.40)	2.22(1.52)	1.44(0.87)	<.0001
**Physical activity**												
YES	59.61	60.4	52.91	NS^2^	68.76	63.06	63.28	NS^2^	45	56.98	42.76	NS^2^
NO	40.38	39.59	47.09		31.25	36.94	36.72		55	43.02	57.24	
**parent BMI**												
father	23.7(2.6)	24.4(2.7)	23.7(2.5)	0.002	23.8(2.7)	24.5(2.8)	23.9(2.6)	0.03	23.6(2.5)	24.3(2.5)	23.3(2.4)	NS
mother	21.6(2.4)	22.02(2.5)	22.2(2.5)	0.03	21.6(2.4)	22.0(2.5)	21.7(2.5)	NS	21.7(2.4)	22.0(2.5)	23.0(2.2)	0.03
**Dietary intake**												
Energy	1613.2(380.4)	1634.7(379.8)	1675.4(324.2)	NS	1692.9 (387.6)	1731.9(379.4)	1707.5(355.1)	NS	1539.2 (358.3)	1518.3(347.8)	1625.6(270.0)	NS
Carbohydrate	220.9(54.3)	219.3(50.2)	227.4(46.4)	NS	230.8(54.2)	231.0(51.8)	232.0(52.1)	NS	211.7(52.7)	205.3(44.6)	220.4(35.8)	NS
Protein	67.1(47.2)	68.4(19.3)	70.0(16.1)	NS	69.1(17.4)	72.4(18.8)	71.2(14.9)	NS	65.3(63.3)	63.7(19.0)	68.1(18.1)	NS
Fat	53.9(17.2)	55.6(17.4)	55.6(15.9)	NS	56.8(17.8)	59.0(17.2)	56.5(16.6)	NS	51.3(16.2)	51.5(16.8)	54.1(15.0)	NS
Sodium	3556.9(995.3)	3618.9(1003.5)	3806.2(1063.8)	NS	3677.6(992.8)	3862.3(1073.3)	3728.3(927.9)	NS	3444.8(985.4)	3327.3(828.4)	3926.6(1259.3)	0.03

^1.^ N(%): number(%)

^2.^ p-value: chi-square from the 6 data in “Age” and “Physical activity”; total, boys and girls

^3.^ Mean(SD): Mean(standard deviation) of continuous variable

^4.^ p-value: ANOVA p-value

^5.^Obesity classification by BMI percentiles: obesity≥95 percentile, 85 percentile≤overweight<95percentile, normal<85 percentile for Korean children obesity criteria from Korean society of obesity.

^6.^ Units for data; SBP & DBP:mmHg, plasma FBS, HDL, LDL & TG:mg/dL, plasma insulin: uU/ml, BMI:Kg/m^2^, energy & carbohydrate intakes:Kcal/day, protein & fat intakes:g/day, sodium intakes:mg/day.

### Effects of sodium intake on obesity risk in children

**Fig 1:**
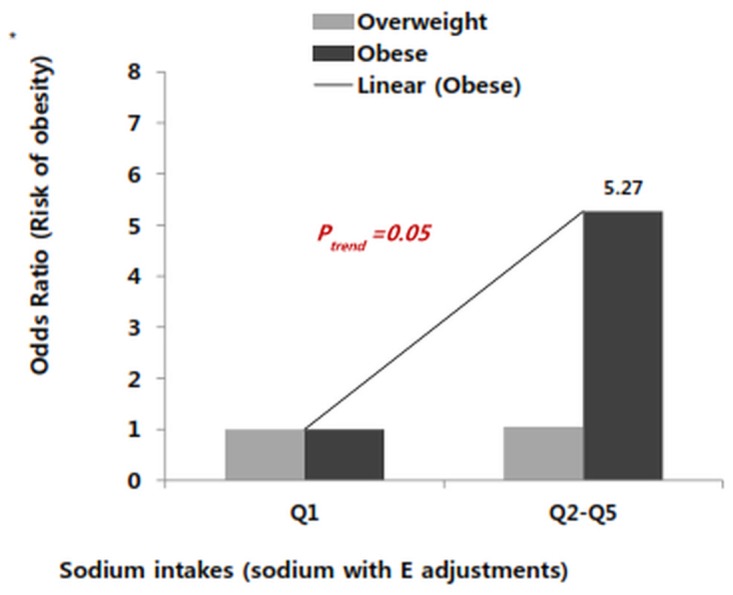
Odds ratio (OR) and 95% confidence interval (CI) for obesity risk by quintiles (Q1-Q5) of sodium intake with energy adjustment (residual-Na) were estimated in total children. The dietary sodium intakes increases, the risk of obesity significantly increases in obesity group (black bar; P-*trend* < 0.05), not in overweight group (gray bar). This figure was shown by comparison with the 1^st^ quintile vs all the others combined. Multivariate logistic regression models after adjustment for age, sex, mother’s BMI, father’s BMI, and education levels (<10 years, 10~12, 13~16 years, and >16 years) of the mother and father, dietary fat, systolic blood pressure and regular physical activity (no, yes). Means of residual-Na in each quintile for total subjects are 2287.6, 2998.9, 3496.8, 4044.9 and 5058.9 mg/day.

Crude sodium intake with no energy adjustment (crude-Na) or sodium intake with energy adjustment (residual-Na) were divided into quintiles, the correlation of overweight with obesity risk was investigated. In the case of the crude-Na intake, the obesity risk was shown to be 6.02-fold higher in the highest sodium intake group (5058.9±619.6 mg/day) than in the lowest sodium intake group (2287.6±347.6 mg/day) in the obesity group. However, the obesity risk did not show linearity with crude-Na intake. (Data not shown) In the case of the residual-Na, the obesity risk was shown to be 2.8-fold higher in the highest sodium intake group than in the lowest sodium intake group within the obese group (p-trend = 0.03). Irrespective of the sex, when the summation of the 2^nd^ (Q2) to 5^th^(Q5) quintile of sodium intake (the means of residual-Na from 4044.9 to 5058.9 mg/day) was compared with the 1^st^ of quintile (Q1), the obesity risk increased 5.27-fold (CI; 1.320–27.560) in the obesity group. (P-trend = 0.05). (Fig. 1) However, the obesity risk was not changed in overweight children according to residual-Na. There was no gender difference in obesity risk by sodium intake was shown because of the difference in the basic sodium intake of the boys and girls (data not shown). From the aforementioned results, children aged between 8–9 years consuming 4000 mg/day or more of sodium showed to have a high risk of obesity.

### Effects of SSG on obesity risk in children

In this study, a total of 10 genes with 11 SNPs were used as SSG such as *ACE*, *ADD1*, *AGT*, *CYP11B2*, *GNB3*, *GRK4 A142V*, *GRK4 A486V*, *NEDD4L*, *SLC 12A3*, *HSD 11β-2*, and *ENac*. However, *HSD 11β-2* and *ENac* SNPs were excluded from this study because their mutants did not exist in our subjects. When the distribution of each SNP genotype (wild, hetero, and mutant) was analyzed in the obese or non-obese children, there was no difference in the distribution of the other SNPs except *GRK4 A486V* was found in the obese children. The *GRK4 A486V* mutant was more distributed in the obese children, whereas the wild genotype was more distributed in the non-obese children (Table 2). Our SSG on the obesity risk were very dependent upon the phenotypes of obesity such as BMI, waist circumferences, obesity index and so on. For examples, the *GRK4 A486V* mutant increased the obesity risk 1.75-fold more when the obesity index was used (P-trend = 0.015), however, the *GRK4 A142V* mutant decreased the obesity risk by 0.7-fold (P-trend = 0.05) when the WC was used (P-trend = 0.058). (Data not shown) These results show that determining the index of obesity risk is very important for investigating the casual relationship of genes related to obesity with the risk factors of obesity.

**Table 2:** The distribution of genotypes for nine SNPs in our children subjects with obesity or non-obesity.

Gene SNP ^1^	Total subjects(%)			Non-Obese Children(%)			Obese Children(%)^3^			p-value ^2^
	Wild	Hetero	Mutant	Wild	Hetero	Mutant	Wild	Hetero	Mutant	
ACE	38.26	47.22	14.52	39.00	46.86	14.14	35.33	48.67	16.00	NS
ADD1	19.52	46.17	34.31	18.58	46.00	35.42	23.26	46.84	29.90	NS
AGT	3.32	30.94	65.74	3.49	31.23	65.28	2.65	29.80	67.55	NS
CYP11B2	10.17	42.66	47.18	9.98	42.81	47.22	10.93	42.05	47.02	NS
GNB3	22.73	52.73	24.53	23.04	52.67	24.29	21.52	52.98	25.50	NS
GRK4 A142V	62.91	32.38	4.7	62.45	32.59	4.96	64.78	31.56	3.65	NS
GRK4 A486V	28.29	51.03	20.68	29.69	50.38	19.93	22.67	53.67	23.67	0.04
NEDD4L	15.35	47.44	37.21	14.70	47.76	37.54	17.94	46.18	35.88	NS
SLC 12A3	83.85	15.49	0.66	84.60	14.82	0.58	80.86	18.15	0.99	NS

^1^ Abbreviation; angiotensin converted enzyme(ACE), angiotensinogen(AGT), α-addicin1(ADD1), cytochrome P450, family 11 subfamily B polypeptide 2(CYP11β-2), G-protein b3 subunit (GNB3), G protein-coupled receptor kinases type 4 (GRK4 A142V, GRK4 A486V), 11β-Hydroxysteroid Dehydrogenase type-2(HSD 11β-2), neural precursor cell-expressed developmentally downregulated 4 like(NEDD4L), solute carrier family 12(sodium/chloride transporters)-member 3(SLC 12A3) and epithelial sodium channel (ENac).

^2^ Significances (ANOVA p-value) on the distribution of nine SNP genotypes in obese (obesity plus overweight) or non-obese (normal) children. NS; not significant.

^3^ Obesity classification by BMI percentiles; obesity≥95 percentile, 85percentile≤overweight <95percentile, normal<85 percentile for Korean children obesity criteria from Korean society of obesity. In this obese group, the overweight group was included.

### Interaction of SSG and sodium intake

**Fig 2:**
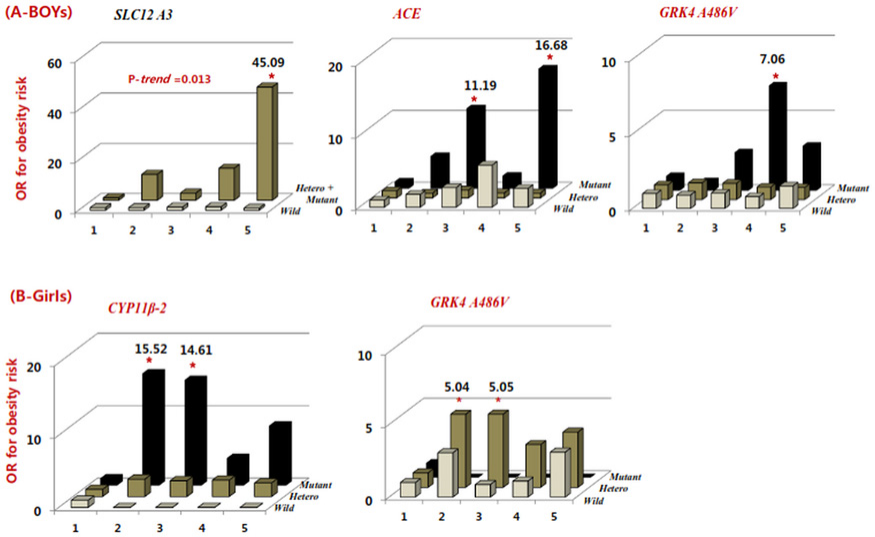
Odds ratio (OR) for obesity risk by quintiles of residual-Na were estimated in boys (A) and girls (B). X-axis means the quintiles of sodium intakes with energy adjustments, Y-axis means OR for obesity risk and bars described. OR in each genotype of gene (lighter; wild homozygote, meddle; heterozygote, darker; mutant homozygote). In case of *SLC12A3*, darker block was described by combination of mutant and hetero because of few mutant frequencies. Statistically significances by p<0.05(*) were shown above bars. Means of the residual-Na in each quintile for boys are 2402.9, 3122.2, 3630.7, 4202.8 and 5194.1 mg/day. Means of residual-Na in each quintile for girls are 2190.8, 2878.4, 3378.9, 3882.5 and 4889.3 mg/day. [Abbreviation: angiotensin converting enzyme; *ACE*, cytochrome P450, family 11 subfamily B polypeptide 2;*CYP11β-2*, G protein-coupled receptor kinases type 4;*GRK4 A486V*, solute carrier family 12(sodium/chloride transporters)-member 3;*SLC12A3*.]

The interaction between SSG and sodium intake showed that the obesity risk 1.0 (reference) was given when the wild type of SSG intake Q1 residual-Na values. We found that OR significantly increased by 7.06, 16.8 and 45.09 fold in the boys with the *GRK4 A486V*, *ACE* and *SLC12A3* mutant as the sodium intake was increased. (Fig. 2-A) OR increased by 45.09-fold (CI; 2.665–762.991) in boys with the highest Na intake (5194.1mg/day) with *SLC12 A3* mutant plus hetero genotypes than in those with the lowest residual-Na with the wild type genotype (P-trend = 0.013). OR increased by 11.19 (CI; 1.475–23.567) and 16.68-fold (CI; 1.234–225.5) in the boys with the *ACE* mutant who had Q3- and Q5-Na intake and 7.06 fold (CI; 1.625–30.646) higher in the boys with *GRK4 A486V* mutant who had Q4-Na intake (4202.8 mg/day) compared to Q1. OR increased by 15.52-fold (CI; 1.816–131.882) and 14.61-fold (CI; 1.623–131.534) among the girls with *CYP11β2* mutant although who had sodium intake about Q2 (2878.4mg/day) or Q3 (3378.9mg/day). Unlike the boys, OR increased approximately 5-fold (CI; 1.166–21.718) more in the girls with the *GRK4 A486V* hetero type who were in low intake of sodium between Q2 and Q3 (2878.4–3378.9mg/day). Therefore, polymorphisms of *GRK4 A486V*, *ACE*, *CYP11Β-2*, and *SLC12A3* showed a gender-based difference in the interaction between the sodium intake and weight gain. (Fig. 2-B) However, the others of SSG were not found the interaction between sodium intake and obesity. It is important to note that confound factors should be carefully determined when screening for obesity genes or SSG.

## Discussion

This was the first Korean children study conducted under the assumption that child obesity is correlated with sodium intake, which were particularly influenced by SSG and gender differences. Compared to the prevalence of obesity in Korean children and adolescence in 1997, it approximately doubled in the amount in 2005 by KNHANES and is likely to further affect the incidence of chronic diseases among adults in the future.[1, 19] As various pediatric obesity criteria, such as BMI, obesity index and WC, have been suggested, the determination of the cutoff of these criteria is controversial in terms of epidemiology or nutrition. However, the percentile-based classification of BMI seems to be a global trend to determine the obesity reference, including for children, as shown in other studies.[20,21] Many reports supported that excessive energy intake, including that of saturated fats, soft drinks, fast foods, and processed foods, have drawn attention as dietary habits affect child obesity.[21] Our results showed that obesity was not correlated with the total intake for energy, sugar, protein and fats, but correlated with sodium intake even in girls. Due to the fact that energy-dense foods contain high sodium, these results implicated that other environmental factors might be involved, such as genetic factors. Sodium intakes among Koreans have increased to approximately 15% from 4036 mg/day in 1998 to 4877.5 mg/day in 2010. According to KNHANES, the sodium intake of non-obese children aged 8 to 9 years was 3446mg for boys and 2928.8mg for girls in 2007 and 2008 and same age in obese children took approximately 1000mg/day more than those found among the normal.[1] Our results demonstrate that high sodium diet, rather than high-energy density diet, was a strong factor in causing obesity in children, particularly in girls. The possibility that high sodium in diet without high-energy food intake may be explained by Korean dietary habits and that rice and Korean soup, with more than 700 mg sodium per one serving size, are commonly combined in every meal. Compared to the lowest residual-Na(Q1), obesity risk with highest residual-Na(Q5) was 1.8 for boys and 2.2 for girls. Similarly, results showed an increase in sodium intake to be proportional to the obese status and the obesity risk was shown to be 6.09-fold higher in the Q5 than in the Q1. As the pattern found in our study is similar to KNHANES, the obese children are likely to consume more sodium in the future. KNHANES also reported that the obesity risk significantly increased in subjects aged 19 years or higher in proportion to their sodium intake.[1] Therefore, a high sodium intake may closely be associated with obesity, asthma, kidney stones, osteoporosis, and gastric cancer among children and adolescence.[22] Similar to other studies, BP, insulin, LDLc, and TG/HDLc ratio significantly increased, however, the HDL proportionally decreased with obesity. [23] It has been well known that hypertension is caused by an increase in BP and body volume due to the accumulation found in the adipose tissues.[24] However, the direct effect of sodium intake on the TG accumulation and proliferation of adipose cells have not been conducted. Several epidemiologic studies have shown that the co-existence of hypertension and diabetes or obesity is due to common genetic and environmental factors such as diet, physical activity and age.[25] However, in obese girls with high sodium intake, sodium intake may be an independent risk factor of obesity-induced insulin resistance independent of BP, because BP and blood insulin, HOMA-IR, TG and LDL were not increased. Although candidate genes related to BP and hypertension have been identified by many researchers, their mechanisms have not yet been established.[26] Environments for BP such as sodium diet, dyslipidemia, BMI and low activity are closely associated with the relevant genes. Thus, the aforementioned factors were adjusted to eliminate their potential influences where the effect of the SSG SNPs on the independent occurrence of pediatric obesity was then investigated.

The obesity risk increased in the boys with the *GRK4 A486V*, *ACE* and *SLC12A3* mutant and in girls with *CYP11β-2* mutant and *GRK4 A486V* hetero type as they increased the residual-Na. The polymorphisms of *ACE*, *SLC12A3 and CYP11β-2* showed a gender difference in the interaction of sodium intakes and obesity. Our previous study found that the association between the *ACE I/D* polymorphism and obesity in relation to sodium intake is gender-dependent in children. Girls with *D* carrier and high sodium intakes exhibited a significant association, whereas *D* carrier boys did not. [27]The result of this study is meaningful in that gender, age or levels of sodium intakes affect obesity as confounding factors in screening for obesity genes or SSG. A Chinese Mongolian study reported a significant interaction between the *CYP11β-2C(-344)T* polymorphism and alcohol consumption on the risk of hypertension.[28] A genome-wide association study showed that *SLC12A3 Arg913Gln* may contribute to genetic susceptibility to diabetic nephropathy among the Japanese.[29] In northwest China, there was no association between the *Arg904Gln* and *Thr418Ser* in *SLC12A3* and hypertension.[30] Even though the association between *SLC12A3* and obesity was not found in this study, the mutation of *SLC12A3* may indirectly increase susceptibility to diabetes, essential hypertension and obesity.[31] Several gene variants are associated with salt sensitivity, but only variants of *GRK4 A486V* have been shown to be highly associated with salt sensitivity in both boys and girls with hetero or mutant genotypes as their residual-Na were increased. Many studies have reported that *GRK4* gene variants were associated with an increase salt sensitivity or with an decrease renin hypertension and sodium excretion because it plays a key role in counter-regulation between the dopaminergic and renin–angiotensin aldosterone systems (RAAS) in the renal proximal tubule, which is the site of approximately 70% of the total renal sodium reabsorption.[14, 32, 33] In particular, a genetic model based on *GRK4 R65L*, *GRK4 A142V and GRK4 A486V* was 94.4% predictive of salt-sensitive hypertension in humans. As well, 2-locus of *GRK4 A142V* and *CYP11β-2 C344T* was 77.8% predictive of low-renin hypertension in Japanese.[14] In terms of the *GRK4* gene, the *A486V* mutant was a risk factor of obesity, whereas the *A142V* mutant suppressed the obesity risk. However, it was interesting to identify gender differences of the *GRK4 A486V* polymorphism on the risk of obesity, and observe that boys who carried mutant homozygotes showed high risks of obesity, while girls carrying heterozygotes, and not mutants, showed high risks of obesity. As a first suggestion, obesity accumulated by fat impaired the insulin metabolism or insulin secretion, independent of BP in obese girls. Dopamine D1A receptor function is impaired in both of obesity-induced insulin resistance and *GRK4* variants, contributing to salt-sensitive hypertension and essential hypertension. [4,34] Since salt-sensitive hypertension and hyperinsulinemia were not found in obese girls as sodium intake was increased, dopamine D1A receptor function was not changed in girls who had GRK4 variants as sodium intake increased. Second, many research reported that the *GRK4 A486V* polymorphism affected the risk of obesity associated with sodium intake, according to how other genes were combined to *GRK4 A486V* variants. In Korean children, the best combination to predict obesity was *SLC12 A3*, *ACE* and *GRK4 A486V* in boys, and *CYP11β-2* and *GRK4 A486V* in girls as sodium intake was increased. Even though we did not examined whether possible haplotypes of those genes increased the risk of obesity, there exist potential obesity risks in boys and girls when they possess different combinations of haplotypes according to high sodium intake.

The mechanisms of the association of genes related to the sodium intake with obesity have not been elucidated yet. One possible explanation is the inflammatory cytokines such as IL-6 resulting from the excess fat may cause the body to retain more sodium and fluid, and elevate BP by activating *ENaC*.[9] This gives motive for physicians to prescribe antihypertensive or anti-obesity therapeutic medicine such as statin or angiotensin II antagonists as they are known to inhibit the secretion of inflammatory factors.[35] The second possibility is the increased ability to incorporate glucose into lipids was observed in isolated adipocytes from high sodium diet tested on rats. Interestingly, a diet high in sodium induced higher adiposity characterized by high plasma leptin concentration and adipocyte hypertrophy, most likely due to an increased lipogenic capacity of WAT.[10] Leptin resistance resulted in an increased salt sensitivity that is mediated by endothelin in the SHHF rat without changes in NOS expression.^6^ Furthermore, the regulators of urinary sodium excretion by AMPK and an energy regulator may be related to obesity related salt sensitivity.[9,10] However, the suppression of AMPK phosphorylation leads to a decrease in sodium excretion and may enhance the salt sensitivity.

We faced many difficulties in this study: first, we assessed a 3-day dietary recall using CanPro SW to evaluate the dietary sodium intake instead of the 24hr urinary excretion of sodium because it was unable to detect in young children with their parents’ permission. Second, we missed the diagnosis of salt sensitivity and could not determine if the subjects had salt sensitive hypertension, or if they were salt sensitive normotensive subjects, due to the uncertain and high-cost methods, low compliance on restricted diets, and unavailability in children. Third, this study was designed for 3 or 6 years cohort study to predict child obesity as who had a mutant of aforementioned genes. However, these results were just shown at the 1^st^ basic status to find what kinds of SSG increase obesity. For example, boys with *GRK4 A486V*, *ACE*, and *SLC12A3* mutants and girls with *GRK4 A486V* hetero and *CYP11β-2* mutant may reduce their daily sodium intake as if they want to reduce the prevalence of obesity. We will show the risk genes of obesity as we finish the cohort study.

## Supporting Information

S1 Table The primers and methods used for detection of SNP of salt sensitivity genes. (DOCX)

## References

[1] Republic of Korea: Ministry of Health & Welfare 2010 Korean National Health and Nutrition Examination Survey (KNHANES); 2010. Available:https://knhanes.cdc.go.kr/

[2] Intersalt Cooperative Research Group, INTERSALT: an international study of electrolyte excretion and blood pressure. Results for 24 hour urinary sodium and potassium excretion. Br Med J 1988;297: 319–328 3416162 10.1136/bmj.297.6644.319 PMC1834069

[3] Weinberger MH. Salt sensitivity of blood pressure in humans, Hypertension 1996;27(3):481–490 8613190 10.1161/01.hyp.27.3.481

[4] Felder RA, White MJ, Williams SM, Jose PA. Diagnostic tools for hypertension and salt sensitivity testing, Curr Opin Nephrol Hypertens.2013; 22(1): 65–76. doi: 10.1097/MNH. 0b013e32835b3693 23197156 10.1097/MNH.0b013e32835b3693 PMC3724405

[5] Chen J, Gu D, Huang J, Rao DC, Jaquish CE, Hixson JE, et al Metabolic syndrome and salt sensitivity of blood pressure in non-diabetic people in China: a dietary intervention study. Lancet. 2009;373: 829–835. 10.1016/S0140-6736(09)60144-6 19223069 PMC2822441

[6] Brown CD, Higgins M, Donato KA, Rohde FC, Garrison R, Obarzanek E, et al Body Mass Index and the Prevalence of Hypertension and Dyslipidemia. Obes Res.2000;8: 605–619. 11225709 10.1038/oby.2000.79

[7] Radin MJ, Holycross BJ, Hoepf TM, McCune SA. Increased salt sensitivity secondary to leptin resistance in SHHF rats is mediated by endothelin. Molecular and Cellular Biochemistry 2003;242: 57–63 12619866

[8] Calson SH, Shelton J, White CR, Wyss JM. Elevated sympathetic activity contributes to hypertension and saltsensitivity in diabetic obese Zucker rats. Hypertension.2010;35: 403–408 10.1161/01.hyp.35.1.403 10642332

[9] Guo D, Zhu H, Hering-Smith KS, Hamm LL, Ouyang J, et al Interleukin-6 stimulates epithelial sodium channels in mouse cortical collecting duct cells. Am J Physiol Regul Intergr Comp Physiol.2010; 299(2): R590–595. 10.1152/ajpregu.00207.2009 PMC2928617 20504903

[10] Fonseca-Alaniz MH, Brito LC, Borges-Silva CN, Takada J, Andreotti S, Lima FB. High dietary sodium intake increases white adipose tissue mass and plasma leptin in rats. Obesity 2007;15:2200–2208. 17890487 10.1038/oby.2007.261

[11] Deji N, Kume S, Araki SI, Isshiki K, Araki H, Chin-Kanasaki M, et al Role of angiotensin II-mediated AMPK inactivation on obesity-related salt-sensitive hypertension. Biochemical and Biophysical Research Communications.2012;418: 559–564. 10.1016/j.bbrc.2012.01.070 22293193

[12] Poch E, González D, Giner V, Bragulat E, Coca A, Sierra A. Molecular Basis of Salt Sensitivity in Human Hypertension Evaluation of Renin-Angiotensin- Aldosterone System Gene Polymorphisms. Hypertension 2001;38:1204–1209. 11711524 10.1161/hy1101.099479

[13] Morrison AC, Boerwinkle E, Turner ST, Ferrell RE. Genome-wide linkage study of erythrocyte sodium-lithium countertransport. Am J Hypertens. 2005;18: 653–6. 15882547 10.1016/j.amjhyper.2004.11.030

[14] Sanada H, Yatabe J, Midorikawa S, Hashimoto S, Watanable T, Moore JH, et al Single-nucleotide polymorphisms for diagnosis of salt-sensitive hypertension. Clinical Chemistry 2006;52(3): 352–360. 16439609 10.1373/clinchem.2005.059139

[15] Kim TH, Hong JM, Oh B, Cho YS, Lee JY, Kim HL, et al Association of polymorphisms in the Interleukin 23 receptor gene with steonecrosis of femoral head in Korean population. Experimetal and molecular medicine. 2008;40:418–426. 18779654 10.3858/emm.2008.40.4.418 PMC2679277

[16] Olson JE, Ingle JN, Ma CX, Pelleymounter LL, Schaid DJ, Pankratz S, et al A comprehensive examination of CYP19 variation and risk of breast cancer using two haplotype-tagging approaches. Breast Cancer Res Treat 2007;102: 237–247. 17004113 10.1007/s10549-006-9324-7 PMC2868324

[17] Han JY, Lim HS, Shin ES, Yoo YK, Park YH, Lee JE, et al Influence of the organic anion-transporting polypeptide 1B1 (OATP1B1) polymorphisms on irinotecan-pharmacokinetics and clinical outcome of patients with advanced non-small cell lung cancer. Lung Cancer 2008;59: 69–75 17766002 10.1016/j.lungcan.2007.07.019

[18] Willett W, Stampfer M. Epidemiology (2nd ed): Implications of total energy intake for epidemiologic analysis. New York: Oxford University Press 1998;273–301 p.

[19] Park JK, Hilmers DC, Mendoza JA, Stuff JE, Liu Y, Nicklas TA. Prevalence of metabolic syndrome and obesity in adolescents aged 12 to 19 Years: comparison between the united states and Korea. J Korean Med Sci. 2010;25(1): 75–82. 10.3346/jkms.2010.25.1.75 20052351 PMC2800028

[20] Seo JY, Cho YG, Kang JH, Hur YI, Park HA, Kim KW, et al New diagnostic criteria for obesity and overweight in Korean children and adolescents using 2007 Korean National Growth Charts. Obesity Research & Clinical Practice. 2013;7(3): e182–189. 23697586 10.1016/j.orcp.2011.12.001

[21] Park S, Lee S, Kim SM, Lee M. Gender specific effect of major dietary patterns on the metabolic syndrome risk in Korean pre-pubertal children. Nutrition Research and Practice 2013;7(2): 139–145. 10.4162/nrp.2013.7.2.139 23610607 PMC3627931

[22] He FJ, Marrero NM, MacGregor GA. Salt intake is related to soft drink consumption in children and adolescents: a link to obesity? Hypertension 2008;51: 629–634. 10.1161/HYPERTENSIONAHA.107.100990 18287345

[23] Kopelman PG. Obesity as a medical problem. Nature 2000;404:635–643. 10766250 10.1038/35007508

[24] Brown CD, Higgins M, Donato KA, Rohde FC, Garrison R, Obarzanek E, et al Body mass index and the prevalence of hypertension and dyslipidemia. Obes Res 2000;8:605–619. 11225709 10.1038/oby.2000.79

[25] Ramachandran V, Ismail P, Stanslas J, Shamsudin N. Analysis of renin-angiotensin aldosterone system gene polymorphisms in malaysian essential hypertensive and type 2 diabetic subjects. Cardiovascular Diabetology 2009;8:11 10.1186/1475-2840-8-11 19243623 PMC2656464

[26] Delles C, Padmanabhan S. Genetics and Hypertension: Is It Time to Change My Practice? Canadian Journal of Cardiology 2012;28: 296–304. 10.1016/j.cjca.2012.02.004 22482397

[27] Yang SJ, Kim S, Park H, Kim SM, Choi KM, Lim Y, et al Sex-dependent association between angiotensin-converting enzyme insertion/deletion polymorphism and obesity in relation to sodium intake in children. Nutrition. 2013;29: 525–530. 10.1016/j.nut.2012.09.001 23398920

[28] Pan XQ, Zhang YH, Liu YY, Tong WJ. Interaction between the C (-344)T polymorphism of CYP11B2 and alcohol consumption on the risk of essential hypertension in a Chinese Mongolian population. European Journal of Epidemiology.2010;25(11):813–821. 10.1007/s10654-010-9504-y 20878543

[29] Kim JH, Shin HD, Park BL, Moon MK, Cho YM, Hwang YH, et al SLC12A3 (Solute Carrier Family 12 member [sodium/ chloride] 3) polymorphisms are associated with end-stage renal disease in diabetic nephropathy. Diabetes.2006;55: 843–848. 16505253 10.2337/diabetes.55.03.06.db05-1013

[30] Wang XF, Lin RY, Wang SZ, Zhang LP, Qian J, Lu DR, et al Association study of variants in two ion-channel genes (TSC and CLCNKB) and hypertension in two ethnic groups in Northwest China. Clin Chim Acta. 2008;388(1–2): 95–98. 17997379 10.1016/j.cca.2007.10.017

[31] Tanaka N, Babazono T, Saito S, Sekine A, Tsunoda T, Haneda M, et al Association of solute carrier family 12 (sodium/chloride) member 3 with diabetic nephropathy, identified by genome-wide analyses of single nucleotide polymorphisms. Diabetes 2003;52: 2848–2853. 14578305 10.2337/diabetes.52.11.2848

[32] Felder RA, Jose PA. Mechanisms of Disease: the role of GRK4 in the etiology of essential hypertension and salt sensitivity. Nature Reviews Nephrology 2006;2: 637–650. 17066056 10.1038/ncpneph0301

[33] Speirs HJ, Katyk K, Kumar NN, Benjafield AV, Wang WY, Morris BJ. Association of G-protein-coupled receptor kinase 4 haplotypes, but not HSD3B1 or PTP1B polymorphisms, with essential hypertension. J Hypertension. 2004;22: 931–936. 10.1097/00004872-200405000-00014 15097232

[34] Trivedi M, Lokhandwala MF. Rosiglitazone restores renal D1A receptor-Gs protein coupling by reducing receptor hyperphosphorylation in obese rats, American J Physiololy. Renal Physiololy 2005;289: F298–F304. 15798088 10.1152/ajprenal.00362.2004

[35] Calò LA, Facco M, Davis PA, Pagnin E, Maso LD, Puato M, et al Endothelial progenitor cells relationships with clinical and biochemical factors in a human model of blunted angiotensin II signaling. Hypertension Research 2011;34: 1017–1022. 10.1038/hr.2011.72 21654754

