# Supplementary material for: Gender-Based Differences on the Association between Salt-Sensitive Genes and Obesity in Korean Children Aged between 8 and 9 Years
Source: PLoS One. 2015 Mar 13;10(3):e0120111. doi: 10.1371/journal.pone.0120111 (PMC4358955; doi:10.1371/journal.pone.0120111)
Supplement: S1 Table — (DOCX) [file pone.0120111.s001.docx]

**S1 table . The primers and methods used for detection of SNP of salt sensitive genes.**

| **No.** | **Gene** | **SNPname** | **rs number** | **Primer sequence** | | **Method** |
| --- | --- | --- | --- | --- | --- | --- |
| 1 | ACE | - | rs4341 | Forward Primer | CTGGAGAGCCACTCCCATCCTTTCT | UHT |
|  |  |  |  | Reverse Primer | GAYGTGGCCATCACATTCGTCAGAT |  |
|  |  |  |  | Genotyping Primer | GGTGAGCTAAGGGCTGGAGCTCAAG |  |
| 2 | ADD1 | G460W | rs4961 | Forward Primer | AGAAGACAAGATGGCTGAACTC | SNaPshot |
|  |  |  |  | Reverse Primer | TAATGTTCGTCCACACCTTAGTC |  |
|  |  |  |  | Genotyping Primer | GACTTGGGACTGCTTCCATTCTGCC |  |
| 3 | AGT | M235T | rs699 | Forward Primer | ATGTTGCTGCTGAGAAGATT | UHT |
|  |  |  |  | Reverse Primer | AGAGAGGTTTGCCTTACCTT |  |
|  |  |  |  | Genotyping Primer | gCggTAggTTCCCgACATATggATggAAgACTggCTgCTCCCTgA |  |
| 4 | CYP11B2 | C-344T | rs1799998 | Forward Primer | tttgatcaattttgcaatga | UHT |
|  |  |  |  | Reverse Primer | AGGGCTGAGAGGAGTAAAAT |  |
|  |  |  |  | Genotyping Primer | AgggTCTCTACgCTgACgATaaagtctattaaaagaATCCAAggC |  |
| 5 | GNB3 |  | Rs5443 | Taqman primer | C_2184734_10 | TagMan |
| 6 | GRK4 | A142V | rs1024323 | Forward Primer | AGCCCCTTTACCAGAAATAC | UHT |
|  |  |  |  | Reverse Primer | GTCTGTTTTGGGGTTAGTCA |  |
|  |  |  |  | Genotyping Primer | AgCgATCTgCgAgACCgTATAAggAggAgAACCCTTCCAAAAAAg |  |
| 7 | GRK4 | A486V | rs1801058 | Forward Primer | GGAGCTGAGAATTGCTGTAG | UHT |
|  |  |  |  | Reverse Primer | TAGCAAACCGAGCATAGAAG |  |
|  |  |  |  | Genotyping Primer | ggATggCgTTCCgTCCTATTgTCCTggATATCgAgCAgTTCTCgg |  |
| 8 | NEDD4L | - | rs2288774 | Forward Primer | CGGTAAGGACAGTCTCATGT | UHT |
|  |  |  |  | Reverse Primer | CCACTTTCCAGAAAGGACTA |  |
|  |  |  |  | Genotyping Primer | ggCTATgATTCgCAATgCTTggggTTTCTAATTgTKTgTAgTCAg |  |
| 9 | SLC12A3 | R904Q | rs11643718 | Forward Primer | CAATAAACCCTCCATGTGTC | UHT |
|  |  |  |  | Reverse Primer | AGCCCCAAAACAGAACTTA |  |
|  |  |  |  | Genotyping Primer | CgTgCCgCTCgTgATAgAATCCAAAACAgAACTTACTgCTCAgCC |  |

**Abbreviation**; angiotensin converted enzyme(ACE), angiotensinogen(AGT), α-addicin1(ADD1), cytochrome P450, family 11 subfamily B polypeptide 2(CYP11β-2), G-protein b3 subunit(GNB3), G protein-coupled receptor kinases type 4 (GRK4 A142V, GRK4 A486V), neural precursor cell-expressed developmentally downregulated 4 like(NEDD4L) and solute carrier family 12(sodium/chloride transporters)-member 3(SLC 12A3).
